# Context, consent, and continuity: Strengthening dementia diagnosis communication

**DOI:** 10.1002/alz.70962

**Published:** 2025-11-24

**Authors:** Filipe Prazeres

**Affiliations:** ^1^ Faculty of Health Sciences, University of Beira Interior Covilhã Portugal; ^2^ Family Health Unit Beira Ria Gafanha da Nazaré Portugal; ^3^ RISE‐Health, Department of Community Medicine Information and Health Decision Sciences, Faculty of Medicine, University of Porto Porto Portugal

**Keywords:** Alzheimer's disease, caregiver burden, community support, disclosure, health communication, informed consent

1

Dear Editor

The study by Singh Solorzano and colleagues^1^ on the psychological impact of revealing a diagnosis of Alzheimer's disease (AD) provides evidence that this event is far from a neutral one. Their finding that nearly half of the studied caregivers developed moderate‐to‐severe post‐traumatic stress symptoms (PTSSs), often persisting for years, underscores the lasting weight of disclosure on caregiver well‐being.^1^ In light of these results, I would like to highlight, from my perspective as a family physician, several aspects of medical communication that deserve closer attention.

First, the characteristics of the space in which disclosure occurred were not directly assessed. Family physicians interact with patients in different health care environments, ranging from their offices to the patient's home and, increasingly, through online platforms,^2^ and since the setting can influence the doctor–patient interaction,^3^ it is important to choose a quiet, private, and well‐lit space and allow enough time for the conversation without external interruptions. In daily practice, we often find ourselves without enough time to dedicate to our patients because of the many tasks at hand. However, for a patient who receives an AD diagnosis, it is essential to show sensitivity to the emotional weight of the diagnosis, a component emphasized in recent guidance,^4^ which, in my experience, cannot be addressed in haste. Singh Solorzano et al.^1^ showed how vividly disclosure moments are remembered, often with intrusive thoughts years later. This raises the question: might the setting itself influence whether disclosure is experienced as traumatic?

Second, the study of Singh Solorzano and colleagues^1^ highlights the Italian context, where diagnoses are often disclosed primarily to caregivers.^1^ This practice, although intended to protect patients, may explain the high levels of distress observed among caregivers. Broader recommendations stress the involvement of both patient and care partner,^4^ but often without clarifying that such involvement must depend on the patient's wishes. Informed, clear, and voluntary consent is a complex issue in persons living with dementia, particularly in stages where they are no longer able to provide free and informed consent.^5^ In these cases, the intervention of a legal representative or court‐appointed guardian is required. The findings of Singh Solorzano and colleagues^1^ remind us that excluding the patient can both undermine autonomy and overload caregivers with responsibility, thereby transforming disclosure into a traumatic event rather than a shared understanding. It is also worth noting that Singh Solorzano et al.^1^ focused on AD disclosure, whereas recent guidance also extends to mild cognitive impairment (MCI).^4^ In MCI, individuals typically retain greater decision‐making capacity, making respect for autonomy and consent even more critical.

Third, Singh Solorzano et al.^1^ concluded that empathic communication and psychological counseling should accompany disclosure,^1^ a crucial step. Yet their results also highlight the need for structured medical follow‐ups and access to community support services, which play a crucial role in helping people with dementia and their careers maintain well‐being and deal with the challenges of the illness,^6^ beyond the initial diagnosis. Although recent guidance has focused on the content of disclosure itself,^2^ the persistence of PTSSs in the Singh Solorzano et al.^1^ study alerts to the risks of leaving families without sustained support after the conversation ends. As family physicians, we often overlook these aspects, but we should not, for the sake of our patients.

Taken together, these observations emphasize that effective communication of an AD diagnosis requires more than clarity and sensitivity in language. It also requires attention to the place where disclosure occurs, whose choices guide involvement, and what services are in place for continuity of care. By integrating these dimensions, physicians may reduce the risk that disclosure becomes a source of enduring trauma and instead make it a constructive step toward shared care planning with the patient, physician, and care partner.

## CONFLICT OF INTEREST STATEMENT

The author has no conflicts of interest to declare. Author disclosures are available in the Supporting Information.

## Supporting information



Supporting Information
